# Supplementary material for: Patterns of Intron Gain and Loss in Fungi
Source: PLoS Biol. 2004 Nov 30;2(12):e422. doi: 10.1371/journal.pbio.0020422 (PMC532390; doi:10.1371/journal.pbio.0020422)
Supplement: Table S1 — Also available at http://genes.mit.edu/NielsenEtAl/. (4.3 MB ZIP). [file pbio.0020422.st001.zip › NielsenEtAl/html/870.html]

AN4016.1.NCU05275.1.MG06044.1.FG01956.1


```
 CLUSTAL W (1.82) Multiple Sequence Alignments - Introns Inserted


Sequence 1: NCU05275.1	129 aa
Sequence 2: FG01956.1	121 aa
Sequence 3: MG06044.1	121 aa
Sequence 4: AN4016.1	131 aa
Alignment Length: 131 aa
Number Identitical Residues: 114 aa
Alignment Score (without introns) 4309


MG06044.1 	----------MTGKTITLEVESSDTIDNVKSKIQDKEGIPPDQQRLIFAGKQLEDGRTLS
NCU05275.1	MVYS--FVKTLTGKTITLEVESSDTIDNVKQKIQDKEGIPPDQQRLIFAGKQLEDGRTLS
FG01956.1 	----------MTGKTITLEVESSDTIDNVKSKIQDKEGIPPDQQRLIFAGKQLEDGRTLS
AN4016.1  	MFFSNSFVKTLTGKTITLDVESSDTIDNVKTKIQDKEGIPPDQQRLIFAGKQLEDGRTLS
          	   :.:  .::*******:*********** *****************************

MG06044.1 	DYNIQK0ESTLHLVLRLRGGIIEPSLKALAS2KFNCDKQICRKCY~ARLPPRA1TNCRKR
NCU05275.1	DYNIQK0ESTLHLVLRLRGGIIEPSLKALAS~KFNCDKMICRKCY0ARLPPRA~TNCRKR
FG01956.1 	DYNIQK0ESTLHLVLRLRGGIIEPSLKALAS~KFNCDKMICRKCY0ARLPPRA~TNCRKR
AN4016.1  	DYNIQK0ESTLHLVLRLRGGIIEPSLKALAS~KYNCEKNICRKCY0ARLPPRA~TNCRKR
          	****** ************************ *:**:* ****** ******* ******

MG06044.1 	KCGHSNQLRPKKKLK
NCU05275.1	KCGHTNQLRPKKKLK
FG01956.1 	KCGHTNQLRPKKKLK
AN4016.1  	KCGHSNQLRPKKKLK
          	****:**********
```
